# Energy criterion guides solvation structure conversion

**DOI:** 10.1093/nsr/nwaf134

**Published:** 2025-04-14

**Authors:** Shi-Gang Sun

**Affiliations:** State Key Laboratory of Physical Chemistry of Solid Surfaces, Department of Chemistry, College of Chemistry and Chemical Engineering, Xiamen University, China

The tiny solvation structure that is formed through interactions between cations, anions and solvents determines the function and properties of electrolytes, and then affects the electrochemical processes. In recent decades, various new electrolyte systems with special solvation structures have been developed toward advanced batteries, while the theoretical study about solvation structure design has been neglected and stagnant, limiting electrolyte development and future applications. Therefore, an appropriate new theory or principle to illuminate the intrinsic nature of the formation and transformation of different electrolyte solvation structures is necessary.

Based on the recognition that bulk electrolyte in a static state is a thermodynamic equilibrium system, Wang *et al.* put forward a novel viewpoint that all electrolytes that contain various solvation structures should follow the same basic thermodynamic laws [1]. The Gibbs free energy, which represents the energy of an electrolyte system, can be considered as a descriptor for judging the change in solvation structures towards more a solvent coordination direction, i.e. free ions, or more anion coordination direction, i.e. aggregations and contact ion pairs. The Gibbs free energy is determined by two aspects: enthalpy and entropy. Enthalpy affects the Gibbs free energy through the thermodynamic competitive equilibrium between cation–solvent interaction and cation–anion interaction. To obtain more negative enthalpy and minimize the Gibbs free energy, stronger cation–anion interactions lead to more aggregation solvation structures, while stronger cation–solvent interactions lead to freer ion solvation structures. Entropy affects the Gibbs free energy through the thermodynamic competitive equilibrium between enthalpy and entropy. To minimize the Gibbs free energy, more negative enthalpy can be achieved by a nearly 100% or 0% ratio trend of one particular solvation structure, while more positive entropy can be achieved by a comparable ratio trend of all kinds of solvation structures. To better guide the design of solvation structures of electrolytes by modifying solvent molecules, solute molecules and their stoichiometric proportion, a unified descriptor ${\vartheta}$ is thus proposed, which refers to the Gibbs free energy discrepancy between a common electrolyte and a new concept electrolyte at the critical state. The critical state means a saturated solute in solvent, as anions coordination has a threshold beyond which the solute precipitates. With the help of the Gibbs free energy criterion determined by enthalpy and entropy, the thermodynamic principle of the solvation structure design in advanced electrolyte systems, such as high-concentration electrolytes, localized high-concentration electrolytes, weak solvated electrolytes, anion coordination electrolytes and high-entropy electrolytes [2–4], can be well explained and unified.

Differently from other research, Wang *et al.* use a thermodynamic pathway to preliminarily discuss and innovate the solvation structure design of electrolytes. This study represents a significant advancement in the theoretical exploration of electrolytes, as recently developed new electrolyte design strategies have been successfully explained by using the thermodynamic competitive equilibrium principle. Based on explicable physical and chemical behaviors, the thermodynamic competitive equilibrium theory/principle proposed by them points out the direction, method and approach for designing more advanced functions of electrolytes, which are beyond the limitations of the uninterpretable predictions that occur when only artificial intelligence is used.

**Figure 1.**
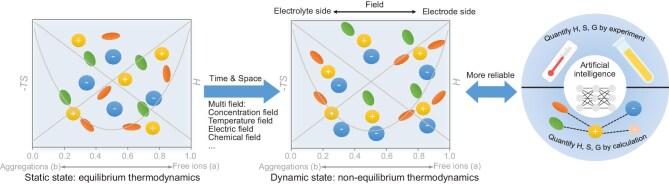
Further development of the thermodynamic competitive equilibrium theory/principle.

Further refinement may be yet needed. For example, equilibrium thermodynamics in Wang *et al.*’s study can only describe and guide electrolyte design at a static state. However, during electrochemical processes, the multitude of fields caused by substance transportation, temperature change, chemical adsorption and other interactions [5] drive electrolytes away from equilibrium and thus the construction of a non-equilibrium thermodynamic model to describe solvation structure change in electrolytes is more accurate and reasonable (Fig. 1). Besides, the synergy between theoretical modeling and experimental validation, such as enthalpy and entropy measurements, theoretical calculation and artificial intelligence analysis, can ensure higher confidence in the reliability of designing electrolytes by using thermodynamics.
